# Unprovoked Stabilization and Nuclear Accumulation of the Naked Mole-Rat p53 Protein

**DOI:** 10.1038/s41598-020-64009-0

**Published:** 2020-04-24

**Authors:** Marian M. Deuker, Kaitlyn N. Lewis, Maria Ingaramo, Jacob Kimmel, Rochelle Buffenstein, Jeff Settleman

**Affiliations:** 1 Calico Life Sciences LLC, South San Francisco, CA USA; 2 Present Address: Oncology R&D Group, Pfizer Worldwide Research and Development, San Diego, CA USA

**Keywords:** Cancer, Tumour-suppressor proteins

## Abstract

The naked mole-rat is a subterranean rodent, approximately the size of a mouse, renowned for its exceptional longevity (>30 years) and remarkable resistance to cancer. To explore putative mechanisms underlying the cancer resistance of the naked mole-rat, we investigated the regulation and function of the most commonly mutated tumor suppressor, *TP53*, in the naked mole-rat. We found that the p53 protein in naked mole-rat embryonic fibroblasts (NEFs) exhibits a half-life more than ten times in excess of the protein’s characterized half-life in mouse and human embryonic fibroblasts. We determined that the long half-life of the naked mole-rat p53 protein reflects protein-extrinsic regulation. Relative to mouse and human p53, a larger proportion of naked mole-rat p53 protein is constitutively localized in the nucleus prior to DNA damage. Nevertheless, DNA damage is sufficient to induce activation of canonical p53 target genes in NEFs. Despite the uniquely long half-life and unprecedented basal nuclear localization of p53 in NEFs, naked mole-rat p53 retains its canonical tumor suppressive activity. Together, these findings suggest that the unique stabilization and regulation of the p53 protein may contribute to the naked mole-rat’s remarkable resistance to cancer.

## Introduction

The naked mole-rat (NMR)is a subterranean mammal residing in the arid and semi-arid grasslands of tropical, northeastern sub-Saharan Africa. Naked mole-rats live in eusocial colonies comprising one breeding female, several breeding males, and a phalanx of subordinate non-breeding workers^1^. They exhibit an exceptionally long lifespan, known to be in excess of 30 years, and show no age-associated exponential increase in risk of dying^2^. This observed maximum lifespan is about five times longer in duration than that predicted by their body mass^3^. The extraordinary longevity of the NMR may reflect, at least in part, their extremely low incidence of cancer^4^. In contrast to mice, where more than 55% of mice die with cancerous lesions identified upon necropsy^5,6^, only a handful of cancer cases have been documented in >2500 necropsies in captive NMRs^7,8^. While long-considered impervious to cancer, recent case reports of carcinogenesis in a few laboratory-housed NMRs do not diminish the fact that NMRs appear to employ remarkable anti-cancer mechanisms that merit further study.

Previously, NMR skin fibroblasts cultured *in vitro* have been shown to display unusual sensitivity to contact inhibition, mediated by induction of p16^INK4A^ and triggered by secretion of high molecular mass hyaluronan^9,10^. Additional studies have demonstrated that the cocktail of large T antigen and oncogenic HRAS^G12V^, a genetic combination that potently induces transformation of mouse and human skin fibroblasts, fails to induce transformation of NMR skin fibroblasts upon implantation in an immunodeficient mouse^11^. Phylogenetic analysis reveals that the NMR genome harbors 17 copies of the phosphatase and tensin (*PTEN*) pseudogene, highlighting the possibility of exceptional regulation of tumor suppressor genes in the NMR^12^. Further, it has been hypothesized that the exceptional proteomic maintenance as well as the constitutively high expression of cytoprotective proteins could promote both the cancer resistance and the exceptional longevity of the NMR^13–15^. However, the precise mechanisms that underlie the NMR’s extreme resistance to cancer remain enigmatic.

Elephants, like NMRs, also display a profound resistance to cancer, putatively resulting from the fact that the elephant genome harbors 20 copies of the tumor suppressor gene *TP53*, several of which are translated into functional protein products that contribute to the DNA damage^16^. While the NMR genome does not encode additional copies of *TP*53, we were intrigued by the possibility that the NMR p53 protein may indeed behave in an unusual manner that contributes to the animal’s cancer resistance.

The importance of p53 in mediating the cancer resistance of elephants comes as no surprise. Standing as the paramount tumor suppressor in the genome, p53 is a multifaceted transcription factor that controls cell cycle arrest, apoptosis, senescence, or DNA repair in response to oncogenic stimuli or loss of genomic integrity^17^. The ability of p53 to rapidly alter the fate of a cell necessitates tight regulation of p53 activity. Under basal conditions in mouse and human cells, p53 is tightly regulated at the protein level via ubiquitin-mediated proteasomal degradation, endowing the protein with a short half-life of approximately 30 minutes^18–20^. DNA damage or an oncogenic stimulus leads to inhibition of the negative regulators of p53, allowing for p53 stabilization, nuclear translocation, tetramerization, and promoter occupancy^21^. p53 is the most frequently mutated gene in human cancer, demonstrating its critical role in protection from the development of cancer^22^.

Canonically, p53 has garnered attention for its role in suppressing cancer, though recent studies investigating enhanced p53 activity illuminate a role for p53 in development and aging as well. Mice carrying one mutant allele of *Tp53* lacking exons 1–6 (*m* allele) display enhanced stability and transactivation activity of the remaining wild-type *Tp53* allele (+ allele), presumably due to p53 C-terminal fragments encoded by the *m* allele augmenting wild-type p53 activities^23^. As expected, augmented p53 activity in *Tp53*^*+/m*^ as compared to *Tp53*^+/−^ mice correlates with resistance to tumorigenesis. Interestingly however, *Tp53*^*+/m*^ mice also display a shorter lifespan than *Tp53*^+/−^ mice, associated with hallmarks of premature aging including tissue atrophy and lordokyphosis (hunchbacked spine). These observations suggest that enhanced p53 activity is not unequivocally advantageous, but rather a double-edged sword preventing cancer while promoting premature aging. In an attempt to circumvent this problem, investigators previously engineered mice to carry a third transgenic allele expressing *Tp53* in its natural genomic context, in addition to the two wild-type *Tp53 *alleles^24^. These “super 53 mice” display remarkable cancer resistance without an early aging phenotype, presumably because in the absence of an oncogenic insult, p53 levels are not elevated relative to mice lacking the transgenic *Tp53* allele.

The phenotype of the NMR stands as a provocative “real life” demonstration of these studies—the NMR displays the cancer resistance characteristic of enhanced p53 activity, coupled with extreme longevity reflecting appropriate p53 regulation. Thus, the centrality of p53 in the complex biology of cancer resistance and longevity motivated us to investigate the functional role of p53 in the NMR, through a combination of biochemical and functional cell-based assays. We found that the NMR p53 protein is far more stable than its murine counterpart, displaying a high level of nuclear localization independent of stressful insult. Despite this basal nuclear localization, NMR p53 nonetheless responds appropriately to DNA damage and activates known tumor suppressor targets identified in other mammals. Thus the unique stabilization and regulation of the p53 protein may contribute to the NMR’s remarkable resistance to cancer.

## Results

### NMR p53 protein displays unusual stability

**Figure 1:**
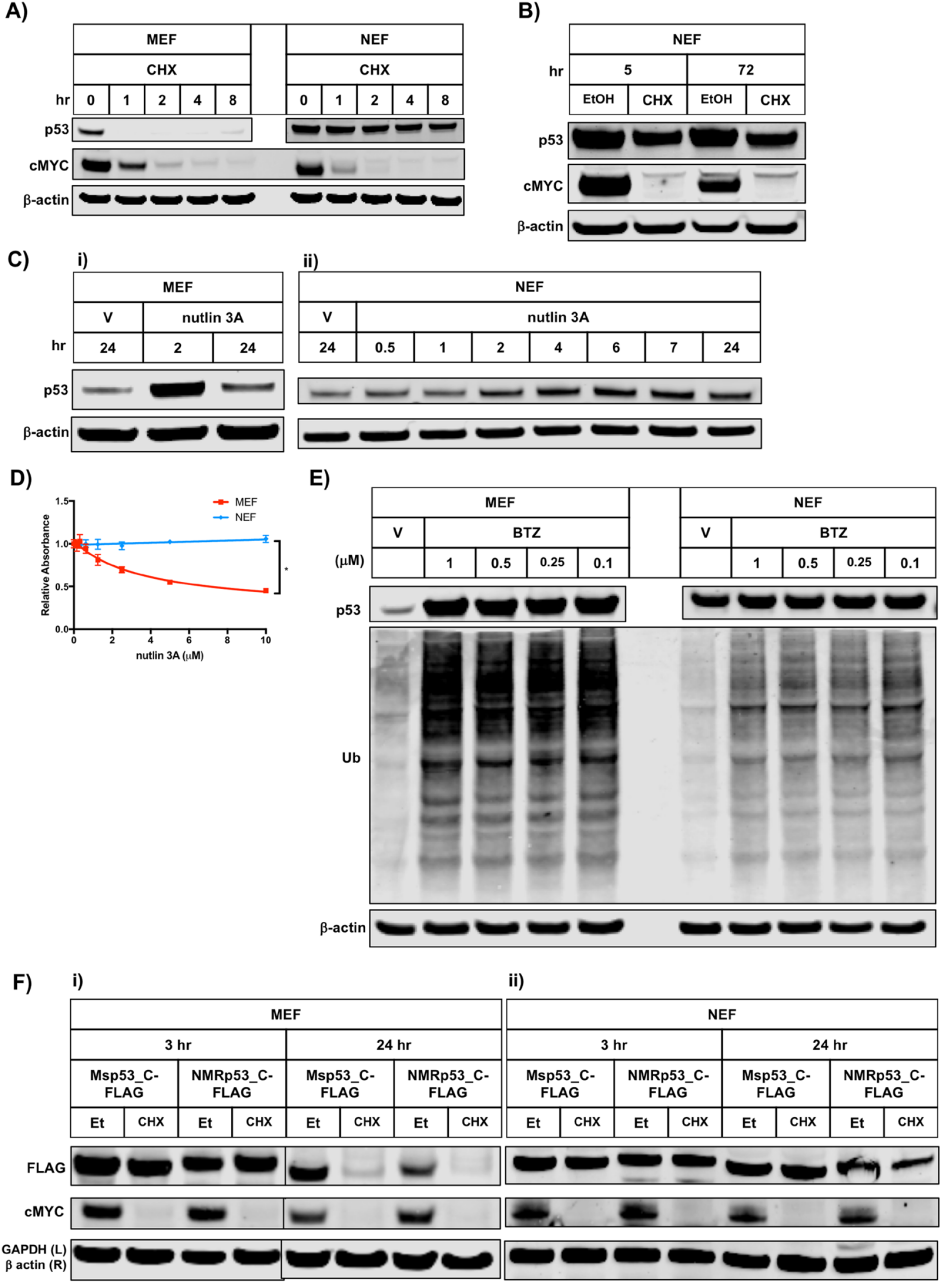
NMR p53 protein displays unusual stability. (**A**) Lysates of mouse embryonic fibroblast (MEF) cells or naked mole-rat embryonic fibroblast (NEF) cells that had been treated for the indicated period of time with CHX (100 μg/mL), were analyzed by immunoblotting with the indicated antisera. (**B**) Lysates of NEF cells that had been treated for the indicated period of time with CHX (100 μg/mL), were analyzed by immunoblotting with the indicated antisera. (**C**) Cells were treated with nutlin 3A (5 µM) or vehicle (V; DMSO) for the indicated time period. Lysates were analyzed by immunoblotting with the indicated antisera. (**D**) MEF or NEF cells were treated with the indicated concentration of nutlin 3A for 72 hours prior to before being fixed and stained with crystal Violet. Crystal Violet staining was quantified by solubilizing the fixed dye and assessing the absorbance at 562 nm. Values are normalized to DMSO control and error bars represent standard deviation. Lines indicate non-linear fit model. Unpaired t test; *p-value < 0.05. (**E**) Lysates of MEF or NEF cells that had been treated for 24 hours with the indicated dose of BTZ (µM) or vehicle (V, DMSO), were analyzed by immunoblotting with the indicated antisera. (**F**) Twenty-four hours after transfection with the indicated p53 construct, MEF (i) or NEF (ii) cells were treated for the indicated period of time with EtOH (Et) or cycloheximide (CHX; 100 μg/mL) prior to harvest. Lysates were analyzed by immunoblotting with the indicated antisera. Parallel black lines in MEF blot images (i) indicate cropping to remove an unnecessary timepoint; however, all MEF samples were run on the same gel.

To investigate the stability of p53 in NMR embryonic fibroblasts (NEFs), we inhibited total protein synthesis with cycloheximide (CHX) and performed immunoblotting at several time points to assess p53 protein levels following translation inhibition (Fig. 1A). For all immunoblots, a monoclonal p53 antibody (DO-1) was used to detect NMR p53 protein, and a polyclonal p53 antibody (FL-393) was used to detect mouse p53 protein (Supp. Table S1). These antibodies were validated extensively via siRNA and CRISPR *Tp53* gene targeting experiments (Supp. Figs. S1 and S2). (We were unable to identify a single antibody that recognized the p53 protein in both mouse and NEFs). As expected^25,26^, in mouse embryonic fibroblasts (MEFs), p53 protein levels reached nearly undetectable levels within one hour of protein synthesis inhibition. By contrast, in NEFs, we failed to detect a significant reduction of p53 protein levels even eight hours after CHX treatment. To confirm the expected protein synthesis inhibitory activity of CHX in NEFs, we also measured levels of cMYC—a protein known to exhibit a short half-life^27^—and observed an appreciable decrease in protein levels within one hour of CHX treatment. Surprisingly, even after 72 hours of CHX-treatment in NEFs, we observed only a modest reduction in NMR p53 protein levels (Fig. 1B).

Given the uncharacteristically long half-life of the NMR p53 protein we sought to investigate whether MDM2, the primary E3-ligase that promotes degradation of p53, also regulates NMR p53. We treated MEFs and NEFs with nutlin 3A, an MDM2 inhibitor that prevents the protein from binding to p53. As expected, in MEFs, nutlin 3A treatment caused a robust increase in p53 levels (Fig. 1Ci). In contrast, in NEFs, treatment with nutlin 3A caused only a minor increase in p53 levels, suggesting that MDM2 may play a less important role in regulating p53 protein levels in NEFs (Fig. 1Cii). Moreover, nutlin 3A treatment reduced the growth of p53 wild-type MEFs but failed to impinge on the growth of p53 wild-type NEFs, further suggesting that MDM2 may play a diminished role in regulating p53 in NEFs as compared to MEFs (Fig. 1D).

Next, we sought to investigate more generally if the proteasome regulates the degradation of NMR p53 protein. We inhibited the proteasome with bortezomib (BTZ) and performed immunoblotting to assess p53 protein levels (Fig. 1E). As expected, in MEFs, p53 protein levels increased robustly upon inhibition of the proteasome^28^. By contrast, in NEFs, even at the highest tested dose of BTZ, we failed to detect an increase in p53 protein levels, demonstrating that inhibition of the proteasome failed to alter p53 protein stability in NEFs. To confirm the expected activity of BTZ in NEFs, we measured bulk levels of ubiquitinated proteins following inhibition of the proteasome and observed an increase in ubiquitinated species upon BTZ treatment.

We hypothesized that two distinct mechanisms could account for the unusual stability of p53 protein in NEFs: 1) The NMR p53 protein exhibits intrinsic stability or 2) Extrinsic regulatory mechanisms within NEFs endow the p53 protein with unusual stability. We anticipated the latter mechanism to be at play, because of the very high degree of homology between the NMR and mouse (as well as human) p53 protein sequences (Supp. Figs. S3A and S3B). In order to disambiguate these two mechanisms, we first measured expression of FLAG-tagged mouse and NMR p53 constructs in their corresponding species-matched fibroblasts, and then performed a “species swapping” experiment, wherein we assessed the stability of mouse p53 expressed in NEFs and of NMR p53 expressed in MEFs (Fig. 1F). Even after 24 hours of translation inhibition with CHX, we observed robust levels of FLAG-tagged mouse and NMR p53 in NEFs (Fig. 1Fii). By contrast, in MEFs, after 24 hours of translation inhibition, we observed nearly undetectable levels of FLAG-tagged mouse and NMR p53 (Fig. 1Fi). Together, these results demonstrate that the unusual stability of NMR p53 derives from a protein-extrinsic, rather than protein-intrinsic, mechanism.

### NMR p53 protein is not stabilized or translocated to the nucleus upon irradiation

**Figure 2:**
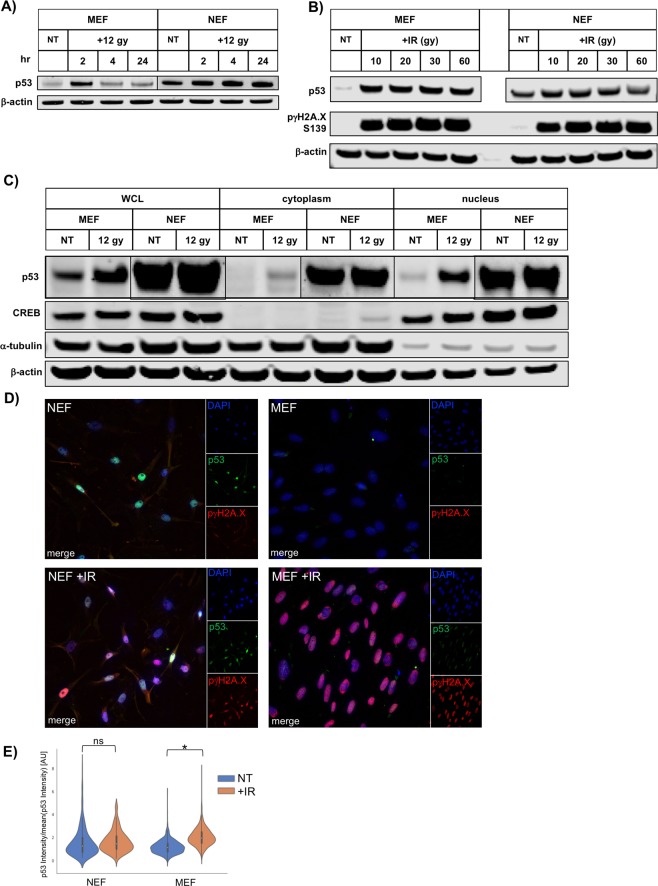
NMR p53 protein is not stabilized or translocated to the nucleus upon irradiation. (**A**) Lysates of MEF or NEF cells, harvested at the indicated time after irradiation (12 Gy), were analyzed by immunoblotting with the indicated antisera. MEF and NEF lysates probed with different p53 antibodies and thus images from two different membranes have been interleaved as indicated at parallel black lines. (**B**) Lysates of MEF or NEF cells, harvested 2 hrs after the dosage of irradiation indicated (Gy), were analyzed by immunoblotting with the indicated antisera. (**C**) Lysates of MEF or NEF cells, harvested 2 hrs after irradiation (12 Gy), were separated into cytoplasmic and nuclear fractions, and analyzed by immunoblotting with the indicated antisera. Whole-cell lysate (WCL). MEF and NEF lysates probed with different p53 antibodies and thus images from two different membranes have been interleaved as indicated at parallel black lines. (**D**) MEF or NEF cells, fixed 2 hrs after irradiation (10 Gy), were stained with the indicated antisera and imaged. (**E**) Quantification of nuclear p53 intensity of images in D). NT indicates no treatment and +IR indicates irradiation (10 Gy). Ns: no significance, *p < 0.05.

The observed lack of change in p53 protein levels in NEFs upon inhibition of either protein synthesis or of the proteasome prompted us to determine if DNA damage, which substantially stabilizes mouse and human p53^29^, could elicit a further stabilization of p53 protein in NEFs. We elected to induce DNA damage via physical stress (irradiation) rather than chemical agents (i.e. chemotherapeutics), to avoid potentially confounding drug metabolism differences between NEFs and MEFs. Bulk p53 protein levels failed to increase appreciably in whole-cell lysates harvested from NEFs post-irradiation (Fig. 2A). By contrast, bulk p53 protein levels increased robustly in whole-cell lysates harvested from MEFs at two hours post-irradiation (Fig. 2A). To confirm that irradiation was indeed inducing DNA damage in NEFs, we assessed levels of DNA damage by measuring phosphorylation of histone H2A.X (Fig. 2B). While ten gray (Gy) was sufficient to induce maximum achievable levels of DNA damage in NEFs, even the highest dose of irradiation tested (60 gy) failed to further stabilize NMR p53 protein levels (Fig. 2B). Interestingly, irradiation induced comparable levels of DNA damage in both MEFs and NEFs.

We then hypothesized that while DNA damage failed to robustly increase bulk p53 levels in NEFs, perhaps DNA damage induced translocation of NMR p53 protein from cytoplasmic sequestration to an active nuclear location. We irradiated NEFs and performed immunoblotting for p53 using whole-cell lysates, or purified cytoplasmic and purified nuclear fractions (Fig. 2C). As expected, in MEFs, this treatment regimen induced nuclear stabilization of p53 protein^30^. In contrast, we observed high levels of nuclear p53 in NEFs before irradiation via immunoblotting, but the level of nuclear p53 (considered to be active p53) failed to increase upon irradiation. To confirm this observation and characterize p53 distribution within individual cells, rather than merely at the population level, we performed immunofluorescence using NEFs fixed under basal growth conditions or post-irradiation (Fig. 2D). We observed considerable heterogeneity of nuclear p53 intensity in NEFs prior to irradiation, but irradiation failed to cause a significant increase in nuclear p53 intensity (Fig. 2E). By contrast, as expected in MEFs, irradiation caused a significant increase in nuclear p53 intensity (Figs. 2D and 2E). Together, these results indicate that DNA damage neither increases the stability of NMR p53 nor induces nuclear translocation of NMR p53 protein.

### NMR p53 protein activates canonical p53 transcriptional targets

**Figure 3:**
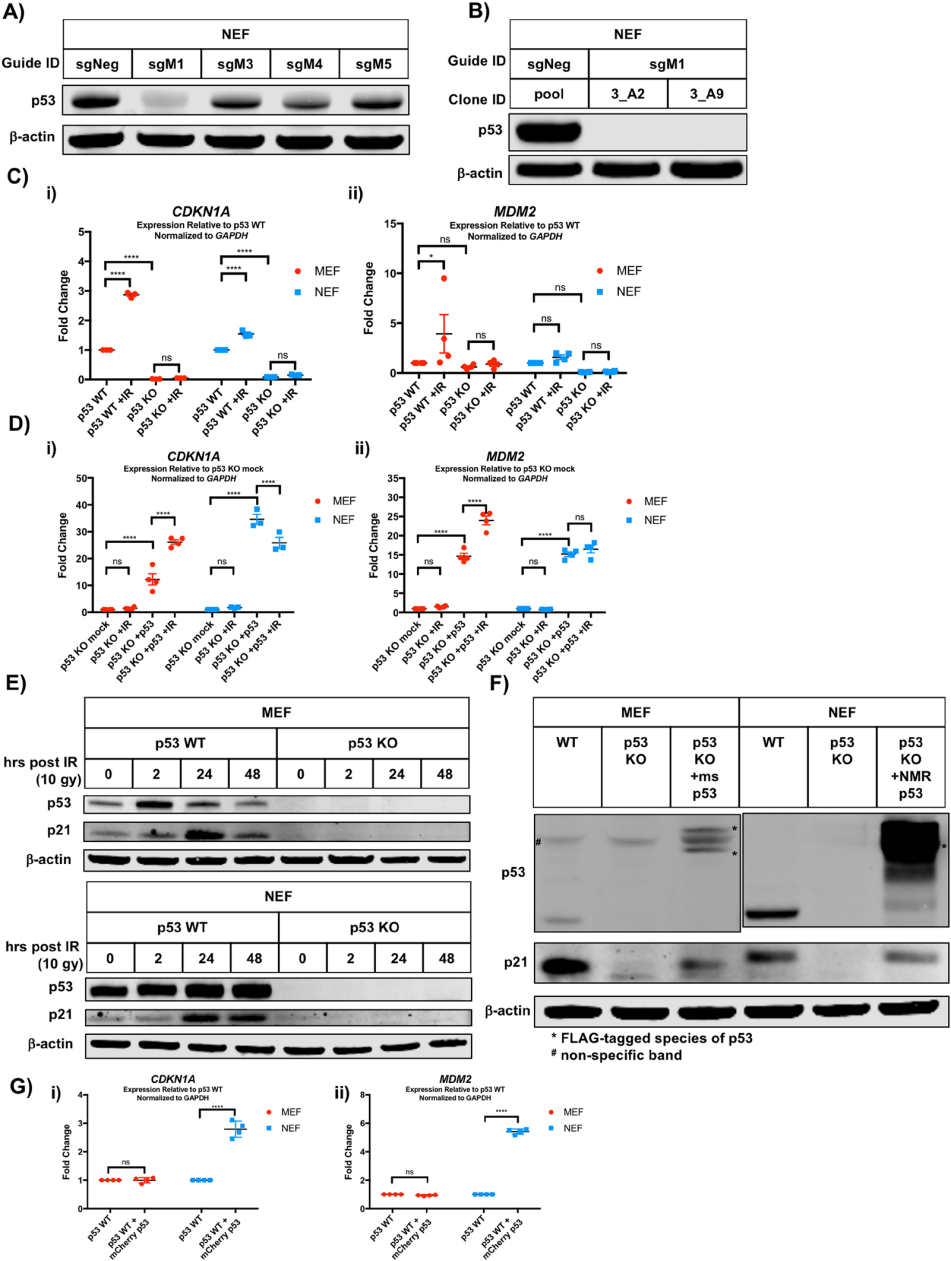
NMR p53 protein activates canonical p53 transcriptional targets. (**A**) Pooled populations of NEF cells, transfected with the indicated guide, were analyzed by immunoblotting with the indicated antisera. (**B**) Single cell-derived clones of NEF cells generated from pooled populations, transfected with the indicated guide, were analyzed by immunoblotting with the indicated antisera. (**C**) Gene expression, presented as fold-change relative to *GAPDH* for *CDKN1A* (i) and *MDM2* (ii), was determined by qRT-PCR at two hours post-irradiation (12 Gy) in MEF and NEF cells of the indicated genotypes. Error bars show SEM. Two-way ANOVA; ****adjusted p-value < 0.0001, *adjusted p-value < 0.05, no significance (ns). (**D**) p53 KO MEF and NEF cells were transfected with p53 rescue constructs, and RNA was harvested 2 hours post-irradiation (12 Gy) for gene expression analysis by qRT-PCR. Error bars show SEM. Two-way ANOVA; ****adjusted p-value < 0.0001, ***adjusted p-value < 0.001, no significance (ns). (**E**) Lysates of MEF or NEF cells, harvested at the indicated time after irradiation (10 Gy), were analyzed by immunoblotting with the indicated antisera. (**F**) Lysates of MEF or NEF cells, transfected with FLAG-tagged p53 as indicated, were analyzed by immunoblotting with the indicated antisera. Parallel black lines in p53 blot indicate MEF and NEF lysates probed with different p53 antibodies. (**G**) Gene expression, presented as fold-change relative to *GAPDH* for *CDKN1A* (i) and *MDM2* (ii), was determined by qRT-PCR in MEF and NEF cells of the indicated genotypes transfected with the indicated constructs. Error bars show SEM. Two-way ANOVA; ****adjusted p-value < 0.0001, *adjusted p-value < 0.05, no significance (ns).

The unusual stability of NMR p53, coupled with its static localization in the face of DNA damage, caused us to question whether the NMR p53 protein acts as a transcription factor to induce expression of canonical p53 target genes in NMR cells. To address this question, we used CRISPR to generate p53 knockout (KO) NEF cell lines. Guide design was influenced by the exon location of guides previously shown to successfully target the mouse *Tp53* gene^31^ (Supp. Fig. S2A and Supp. Table S2). We assessed the level of p53 protein reduction in pooled populations of NEFs transfected with five different guides (Fig. 3A) and observed that cells transfected with guide M1 displayed the most robust reduction in p53 protein. We derived single cells clones from this pooled population, and identified several clones exhibiting complete p53 protein loss (Fig. 3B and Supp. Fig. S2B). In parallel, we derived single cell MEF clones exhibiting complete p53 protein loss, using previously published guides (Supp. Fig. S2C)^31^. As anticipated, the growth inhibitory effect of nutlin 3A on p53 wild-type MEFs (Fig. 1D) was abolished in p53 knockout MEFs, and the growth of p53 knockout NEFs remained unaltered in the face of nutlin 3A treatment (Supp. Fig. S2D). To assess the ability of NMR p53 to induce expression of canonical p53 target genes, we quantified expression of *MDM2* and *CDKN1A* in wild-type and p53 knockout cells under basal conditions, and after irradiation (Fig. 3Ci and Cii). We observed that loss of p53 protein reduced expression of both *MDM2* and *CDKN1A* in NEFs under basal conditions, and also abolished induction of these genes following irradiation. In order to confirm that the loss of p53 target gene expression was a direct result of p53 loss, we performed a rescue experiment wherein we transfected p53 knockout NEFs with a FLAG-tagged NMR p53 construct. As expected, p53 knockout NEFs re-expressing NMR p53 protein displayed a massive induction of *CDKN1A* and *MDM2* expression (Fig. 3D). We performed immunoblotting to confirm these results at the protein level. We observed that loss of p53 in NEFs abrogates irradiation-induced expression of p21 protein (Fig. 3E) and that expression of FLAG-tagged NMR p53 in p53 knockout NEFs rescued p21 protein expression (Fig. 3F). Finally, transient transfection of *Tp53* significantly increased the expression of *CDKN1A* and *MDM2* in wild-type NEFs but not wild-type MEFs, suggesting that perhaps NMR cells lack the ability to suppress the activity of excess p53 (Fig. 3G). Together, these results demonstrate that despite its unusual stability and localization, the NMR p53 protein does indeed act as a transcription factor and induces expression of at least a subset of canonical p53 target genes.

### NMR p53 protein controls genome integrity and behaves as a tumor suppressor

**Figure 4:**
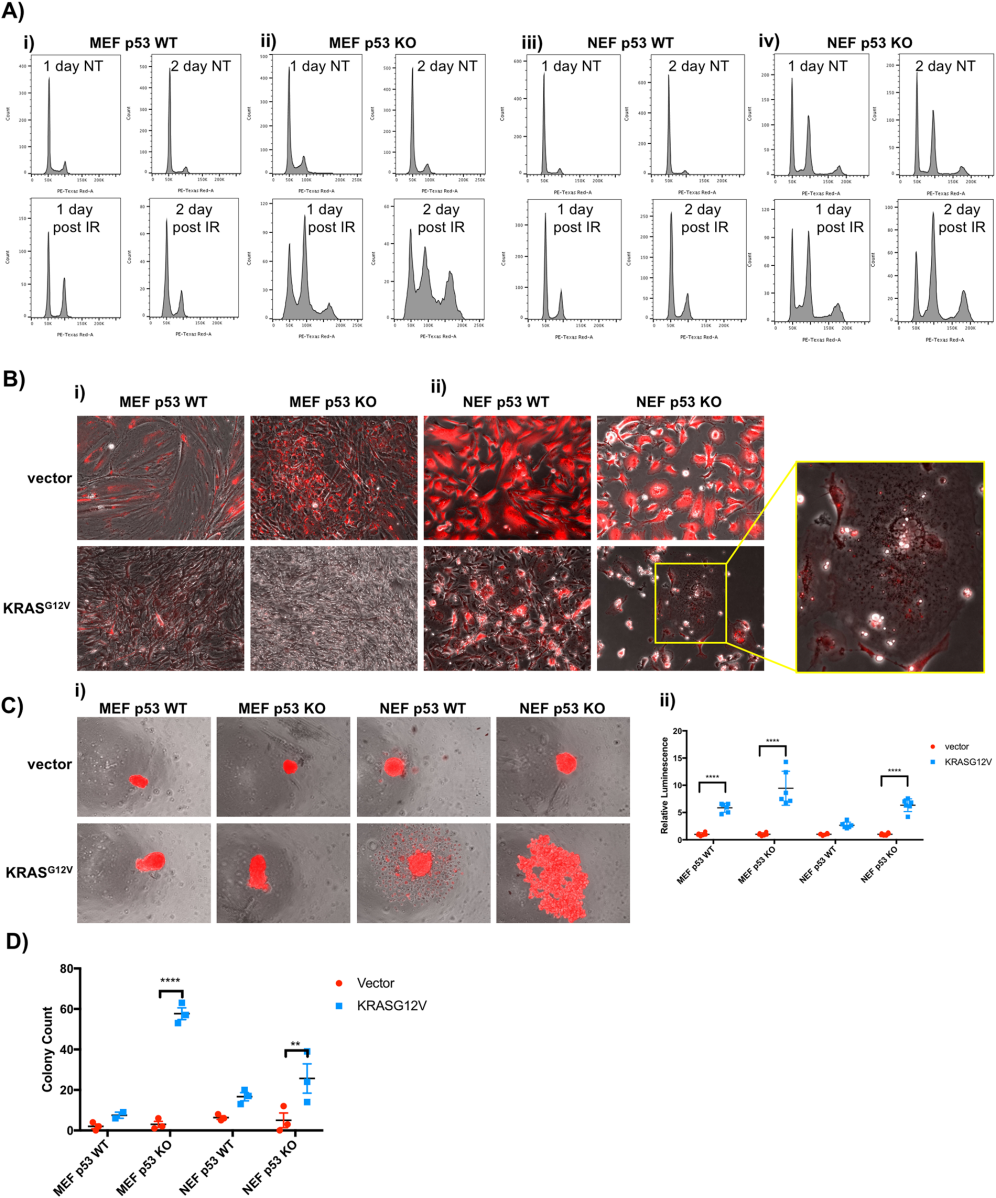
NMR p53 protein controls genome integrity and functions as a tumor suppressor. (**A**) MEF or NEF cells of the indicated genotypes were fixed after irradiation (12 Gy). DNA content was quantified by flow cytometry. (**B**) MEF or NEF cells of the indicated genotypes were infected with constructs expressing either mCherry or an mCherry KRAS^G12V^ fusion protein. Cells were imaged 12 days post-infection. (**C**) MEF or NEF cells of the indicated genotypes were infected with constructs expressing either mCherry or an mCherry KRAS^G12V^ fusion protein. Twenty-four hours post-infection, cells were replated in ultra-low attachment plates. Cells were imaged and quantified 21 days post-infection. Two-way ANOVA; ****adjusted p-value < 0.0001. (**D**) Cells of the indicated genotype were infected with the specified construct and allowed to grow in a soft agar colony assay for 50 days prior to colony quantification. Two-way ANOVA; ****adjusted p-value < 0.0001, **adjusted p-value < 0.01.

After determining that NMR p53 does indeed act as a transcription factor and induces expression of canonical p53 target genes, we sought to investigate if, despite its intransient expression levels, p53 also controls genome integrity in NEFs. First, we examined the ability of p53 wild-type and p53 knockout NEFs to undergo cell cycle arrest post-irradiation. As anticipated by results in MEFs (Figs. 4Ai and 4Aii), we observed an irradiation-induced G2/M arrest in p53 wild-type NEFs (Fig. 4Aiii). Even prior to irradiation, p53 knockout NEFs displayed a greater distribution of cells in the G2 phase than p53 knockout MEFs, and this distribution was greatly increased upon irradiation (Fig. 4Aiv). Moreover, a population of tetraploid cells emerged in the p53 knockout NEFs prior to irradiation, indicating that NMR p53 plays a role maintaining genome integrity even under basal conditions. This aneuploid NEF population expanded further in the p53 knockout cells post-irradiation.

With the knowledge that p53 in NEFs does indeed promote genome integrity, we performed oncogenic transformation studies to determine if p53 loss is required for RAS-mediated transformation of NEFs. First, we investigated morphological effects of KRAS^G12V^ in p53 wild-type and knockout cells. As expected, KRAS^G12V^ expression caused p53 wild-type MEFs to assume a spindly morphology and undergo contact inhibition, whereas the p53 knockout MEFs assumed a transformed morphology upon KRAS^G12V^ expression and grew in an overlapping manner, forming small foci (Fig. 4Bi). By contrast, KRAS^G12V^ expression in p53 knockout NEFs reduced cellular proliferation in two-dimensional cell culture, with the emergence of a small number of large, multi-vacuolated cells (Fig. 4Bii). However, two-dimensional cell culture often fails to capture the nuances of cellular transformation, so we performed three-dimensional cell culture studies to further explore transforming potential. Quantification of spheroid growth in ultra-low attachment (ULA) plates revealed that loss of p53 does indeed promote KRAS^G12V^-mediated anchorage-independent spheroid formation in NEFs (Fig. 4C). Finally, measuring anchorage-independent growth via colony formation in soft agar corroborated the conclusion that p53 protects NEFs from KRAS^G12V^-induced transformation, and demonstrates that loss of p53 is sufficient to allow NEFs to undergo KRAS^G12V^-induced transformation (Fig. 4D). Together, these findings demonstrate that, despite the atypical regulation of p53 expression in NEFs, the protein exerts anti-tumor functions as measured in *in vitro* transformation assays.

## Discussion

The relatively short half-life of p53 has previously been recognized as integral to the protein’s regulation and biological function as a critical tumor suppressor. The perpetual cycle whereby the p53 protein is translated and rapidly degraded under basal conditions is thought to allow a cell to nimbly respond to assaults on genomic integrity by stabilizing pre-existing p53 protein, and thereby promptly initiate DNA repair, cell cycle arrest, or apoptosis^21^. We asked if p53 contributed in a unique manner to the observed cancer resistance of the NMR. Here, we have found that the NMR p53 protein is regulated in a fundamentally different manner. Rather than undergoing rapid degradation-based regulation, we show that in NMR cells in culture, the p53 protein has a half-life greatly exceeding that of other species^18–20^. This observation led us to hypothesize that the NMR spatially sequesters p53 protein prior to the onset of cellular stress, in order to avoid constant inappropriate activation of p53 target genes promoting cell cycle arrest or apoptosis. To our surprise, assessment of p53 localization by both immunofluorescence and cellular fractionation revealed that a significant fraction of p53 protein is constitutively localized in the nucleus of NEFs prior to DNA damage. Thus, our hypothesis of cytoplasmic p53 sequestration proved incorrect. The mechanism regulating suppression of NMR p53 protein activity under basal conditions when the genome of an NMR cell is undamaged remains enigmatic. Future studies interrogating the possibility of NMR p53 undergoing sequestration within a nuclear sub-compartment will prove illuminating. Additionally, investigating post-translational modifications of NMR p53 protein may better explain the protein’s enhanced stability. Sequences alignments reveal the NMR protein retains all lysine residues involved in ubiquitination modifications, but a more detailed exploration is warranted. Finally, a mass spectrometry-based approach promises to elucidate absolute levels of p53 protein in NMRs cells, and perhaps further explain differential regulation of the p53 protein in NMRs compared to previously characterized species.

One attractive explanation for our observation that NMR p53 undergoes nuclear localization prior to the onset of DNA damage is that many binding partners associate with p53 under basal conditions. These putative binding partners may allow for activation of a subset of target genes that are not involved in the canonical p53 directed outcomes of apoptosis, cell cycle arrest, or DNA damage repair. Our gene expression studies demonstrate that DNA damage induces NMR p53 to activate canonical target genes; thus, some level of regulation necessarily prevents this activation prior to DNA damage. Additionally, our observation that transfection of *Tp53* in wild-type cells induces increased expression of p53 target genes in NEFs but not MEFs suggests that perhaps the ability of wild-type NEFs to restrain the activity of p53 is taxed at full capacity, and thus NMR cells lack the additional buffering capacity employed by MEFs to inhibit transcriptional output directed by excess p53. Futures studies investigating p53-interacting partners in the NMR will prove highly informative, as will studies investigating p53 DNA occupancy under basal and stressed conditions.

Given the unexpected nature of p53 protein regulation in NMR cells, we questioned whether the protein has evolved to no longer exert tumor suppressive activity. However, examining the genome integrity of NMR cells in the context of DNA damage and of oncogenic RAS activation revealed that p53 retains its canonical tumor suppressive role. Moreover, p53 knockout NEFs display compromised genome integrity even in basal conditions, suggesting p53 may be present at high levels prior to overt DNA damage as a mechanism of maintaining genome integrity under basal conditions. Ultimately, a fundamental question emerges as we synthesize the observations that NMRs are highly resistant to tumorigenesis and also regulate p53 in a unique manner: how does the unique regulation of naked mole rat p53 protect the organism from carcinogenesis? The knowledge that excess p53 activity promotes aging via uncontrolled activation of apoptosis and senescence further suggests that NMRs necessarily regulate p53 in a unique way to avoid the deleterious aspects of p53 activation.

The cancer-resistant phenotype of the NMR provides an opportunity for an enhanced understanding of mechanisms that protect from tumorigenesis. Our observation that the p53 protein—the major tumor suppressor in the human genome—is subject to fundamentally different regulation in the NMR as compared to all other species studied to date, suggests that elucidation of this atypical regulation may provide unique drug development strategies for cancer therapeutics.

## Methods

### Cell culture

NMR fibroblast lines were generated as previously described^11^. Mouse cell lines were acquired from the American Type Culture Collection. Cell lines were tested for mycoplasma monthly. Cells were cultured at 35°C, 5% CO_2_, 3% O_2_ on collagen I-treated plates (Corning) in minimum essential medium containing Earle’s Salts and L-Glutamine (Gibco) supplemented with 10% fetal bovine serum, penicillin (100 units/mL), streptomycin (100 μg/mL), and Amphotericin B (250 ng/mL).

### Use of animals and ethical statement

Naked mole-rats were housed in captivity with approval from Fish and Wildlife, California and the USDA at the Buck Institute for Research on Aging (Buck), an AAALAC accredited facility. Animals were kept on a 12 h light:dark cycle under conditions simulating natural conditions in the wild (30°C; 50% RH) and maintained on a diet of fresh fruit and vegetables. Tissue and cell harvesting procedures were approved by the Buck IACUC, protocol #10199 and followed the guidelines outlined by the National Institute of Health (NIH) for animal experimentation.

### Immunoblot Analysis

Cells were lysed using radioimmunoprecipitation buffer (25 mM Tris•HCl pH 7.6, 150 mM NaCl, 1% NP-40, 1% sodium deoxycholate, 0.1% SDS; Thermo Fisher) containing protease and phosphatase inhibitors (Thermo Fisher). After a 30 minute incubation at 4°C with occasional vortexing, samples were centrifuged at 4°C for 10 min at 21,130 × g to generate protein lysates. Protein concentration was obtained using the bicinchoninic acid assay (Pierce/Thermo Scientific). Thirty μg of protein were separated using NuPAGENovex 4–12% Bis-Tris gels (Invitrogen) with NuPAGE MES SDS buffer (Invitrogen) and then transferred to a nitrocellulose membrane (Invitrogen) using an iBlot 2 transfer apparatus (Invitrogen). Membranes were blocked for one hour at room temperature in Odyssey blocking buffer (LI-COR Biosciences) and probed overnight at 4°C with primary antibodies (described in Supp. Table S1) diluted 1:10,000 in Odyssey blocking buffer supplemented with 0.1% Tween-20. Membranes were washed with TBST and antigen–antibody complexes were detected using fluorescent secondary antibodies (LI-COR Biosciences) and visualized with a LI-COR infrared imaging system (Odyssey Fc). Cellular fractionation was performed using a Nuclear Extract Kit (Active Motif) as directed by manufacturer’s instructions.

### Transient Transfection

pCMV3-msp53-CFLAG vector was obtained from Sino Biological (ref. seq. NM_011640.3). A gene block of NMR p53 (ref. seq. NP_001297199.1) was obtained from IDT and cloned into the pCMV3 backbone to generate the pCMV3-nmrp53-CFLAG vector. Transient transfections were performed using Lipofectamine 2000 (Invitrogen) and OptiMEM (Invitrogen). Irradiation was performed using the CellRad cabinet x-ray cell irradiator (Precision X-Ray, Inc.) using manufacturer guidelines.

### siRNA Gene knockdown

Cells were transfected with siRNA (Dharmacon) targeting *Tp53* using LipofectamineRNAiMAX and analyzed 72-hours post transfection.

### CRISPR Editing

Guide sequences (Supp. Table S2) were introduced using the Alt-R CRISPR Cas9 system (IDT), following manufacturer’s guidelines. Alt-R CRISPR-Cas9 tracrRNA – ATTO 550 was used to facilitate identification of successfully transfected cells. Ribonucleoprotein complex was delivered via reverse transfection using LipofectamineRNAiMAX (Invitrogen) and Opti-MEM (Invitrogen). Twenty-four hours after transfection, cells were subjected to fluorescence activated cells sorting to enrich for ATTO 550 positive cells (top 40%). Two weeks later, single cells were sorted into 96-well plates. Clonal populations were expanded and tested for loss of p53 protein expression via immunoblotting.

### Gene expression

RNA was isolated from cells using the RNeasy kit (Qiagen) with the QIAcube (Qiagen). Reverse transcription was performed using the High Capacity RNA-to-cDNA Kit (Applied Biosystems). Gene expression assays were performed using the PowerUpSYBR Green Master Mix (Applied Biosystems) and the QuantStudio 6 Flex Real-Time PCR System (Applied Biosystems).

### Cell cycle analysis

All cell cultures were seeded at a density (5×10^5 cells in a 10 cm plate) such that they retained proliferative potential at the time of analysis. Cells were fixed by dropwise addition, while vortexing, of 70% ethanol diluted in water and allowed to incubate at −20°C overnight. Cells were washed in PBS, resuspended in PBS containing RNAseA (0.1 mg/mL) and Triton X-100 (0.05%) for 30 minutes, and then stained with propidium iodide (50 ug/mL) for 15 minutes at 37°C prior to analysis (BD-Fortessa X-20). Data were analyzed using FloJo.

### Colony growth in soft agar

Twenty-four hours after infection with appropriate constructs, cells were suspended in soft agar in 6-cm plates in triplicate. After 50 days colony formation was quantified using the Gelcount (Oxford Optronix).

### Ultra-low attachment assay

Twenty-four hours after infection with appropriate constructs, cells were replated in ultra-low attachment plates (Corning Costar). After 20 days spheroids were visualized and quantified the CellTiter-Glo three-dimensional cell viability reagents (Promega).

### Immunofluorescence

Cells were plated on glass slides and then fixed for 10 minutes at 37°C in 4% paraformaldehyde freshly diluted in PBS. Cells were washed with PBS, blocked for one hour at room temperature in blocking buffer (5% normal goat serum, 0.1% Triton X-100 in PBS), and probed overnight at 4°C with primary antibodies (described in table S1) antibody diluent buffer (1% BSA, 0.1% Triton X-100 in PBS). Cells were washed with PBS, antigen–antibody complexes were detected using fluorescent secondary antibodies (Alexa Fluor 488 and Alexa Fluor 594), counterstained with DAPI, and mounted with ProLong gold antifade (Thermo Fisher Scientific). Wide-field fluorescence images were acquired on a DMI8 (Leica biosystems) using the LAS X software. Excitation was achieved using a SOLA SE-U with DAPI, GFP-T, Cherry-T and Y5-T cubes (Leica biosystems). Fluorescence was collected on a Hamamatsu ORCA-Flash4 camera through a HC PL APO CS 40×/0.85 air objective. FIJI was used to perform image analysis. For experiments measuring p53 localization after irradiation, cell nuclei were segmented using a fully convolutional DenseNet^32^ trained on the Broad Benchmark Collection BBC038v1^33^ and manual annotations. The mean intensity of the p53 channel was measured inside each nucleus.

### Pharmacologic treatments

Cells were treated with the pharmacologic agents as described (Supp. Table S3).

### Cell growth assays

Cells were seeded and treated with pharmacologic agents as described for 72 hours, at which time viable cells were stained with crystal violet and quantified by solubilization in 33% (v/v) acetic acid with A562 absorbance assessed.

### Statistical analysis

For p53 localization experiments, we compared the distribution of mean p53-nuclear intensities in unirradiated cells to the distribution of irradiated cells in each species using a Rank Sums non-parametric test (Fig. 2E). For gene expression (Figs. 3C and 3D) and ultra-low attachment studies and colony formation studies (Figs. 4C and 4D) we performed a two-way ANOVA using Turkey’s multiple comparisons test. For cell growth studies (Fig. 1D and Supp. Fig. 2D) we performed an unpaired t test.

## Supplementary Information

Supplementary Information.
