# Supplementary material for: Unprovoked Stabilization and Nuclear Accumulation of the Naked Mole-Rat p53 Protein
Source: Sci Rep. 2020 Apr 24;10:6966. doi: 10.1038/s41598-020-64009-0 (PMC7181817; doi:10.1038/s41598-020-64009-0)

Unprovoked Stabilization and Nuclear Accumulation of the Naked Mole-Rat p53 Protein

Marian M. Deuker[1], Kaitlyn N. Lewis Hardell[1], Maria Ingaramo[1], Jacob Kimmel[1], Rochelle Buffenstein[1,#], Jeff Settleman[1,2*]

[1.] Calico Life Sciences LLC, South San Francisco, CA, USA

[2.] Current Address:
Oncology R&D Group, Pfizer Worldwide Research and Development,
San Diego, CA, USA

* Corresponding author
Email: jsettleman@gmail.com

[#] Please direct any requests for naked mole-rat related reagents or questions regarding the animals to rbuffen@calicolabs.com

Abstract Word Count: 192

Main Text Word Count (Excluding References and Figure Legends): 3485

Number of Figures: 4

Supplemental Figure Legends

Supplemental Figure S1

A) MEF or NEF cells transfected with siRNA targeting *Tp*53 were analyzed by immunoblotting with the indicated antisera two hours post-irradiation (12 Gy).

Supplemental Figure S2

A) Location of CRISPR guide sequences targeting mouse (i) and NMR (ii) p53, as indicated.
B) Single cell-derived clones of NEF cells generated from pooled populations, transfected with the indicated guide, were analyzed by immunoblotting with the indicated antisera.
C) Single cell-derived clones of MEF cells generated from pooled populations, transfected with the indicated guide, were analyzed by immunoblotting with the indicated antisera two hours post-irradiation (12 Gy).
D) p53 KO MEF or NEF cells were treated with the indicated concentration of nutlin 3A for 72 hours prior to before being fixed and stained with crystal violet. crystal violet staining was quantified by solubilizing the fixed dye and assessing the absorbance at 562 nm. Values are normalized to DMSO control and error bars represent standard deviation. Lines indicate non-linear fit model. Unpaired t test; Ns: no significance.

Supplemental Figure S3

A) NMR and mouse p53 protein sequences were aligned.
B) NMR and human p53 protein sequences were aligned.

Supplemental Figure S4

A) Uncropped membranes from images in figure 1A (i), figure 1B (ii), figure 1C (iii), figure 1E (iv), and figure 1F i (v 1) and 1F ii (v 2). Red boxes indicate lanes included in figure. L indicates left blot and R indicates R blot. DO-1 and FL393 indicate antibody used for p53 detection (see supplementary table S1).

B) Uncropped membranes from images in figure 2A (i), figure 2B (ii) and figure 2C (iii). Red boxes indicate lanes included in figure. DO-1 and FL393 indicate antibody used for p53 detection (see supplementary table S1).
C) Uncropped membranes from images in figure 3A (i), figure 3B (ii), figure 3E (iii), and figure 3F (iv). Red boxes indicate lanes included in figure. DO-1 and FL393 indicate antibody used for p53 detection (see supplementary table S1).
D) Uncropped membranes from images in supplementary figure S1. DO-1 and FL393 indicate antibody used for p53 detection (see supplementary table S1).
E) Uncropped membranes from images in supplementary figure S2A (i) and supplementary figure S2B (ii). DO-1 and FL393 indicate antibody used for p53 detection (see supplementary table S1).

Supplementary Table S1. Antibodies used for immunoblot and immunofluorescence experiments.

Epitope	Company	Clone and Catalog Number	Cell Line Antibody Used In
α-tubulin	Cell Signaling Technology	Clone DM1A; Cat. 3873	Mouse and NMR
β-actin	Cell Signaling Technology	Clone 8H10D10; Cat. 3700	Mouse and NMR
β-actin	Cell Signaling Technology	Clone 13E5; Cat. 4970	Mouse and NMR
c-MYC	Cell Signaling Technology	Clone D3N8F; Cat. 13987	Mouse and NMR
CREB	Cell Signaling Technology	Clone 48H2; Cat. 9197	Mouse and NMR
FLAG	Sigma Aldrich	Clone M2; Cat. F1804	Mouse and NMR
GAPDH	Cell Signaling Technology	Clone D16HL; Cat. 5174	Mouse and NMR
p21	BD Biosciences	Clone SXM30; Cat. 556431	Mouse and NMR
p53	Santa Cruz	Clone D-O1; Cat. SC-126	NMR
p53	Santa Cruz	Clone FL-393; Cat. SC-6243	Mouse (immunoblots)_
p53	Leica Biosystems	Clone CM5; Cat. P53-CM5P-L	Mouse (immunofluorescence)
Phospho-γH2A.X Ser139	Abcam	Clone 9F3; Cat. 26350	Mouse and NMR
Phospho-γH2A.X Ser139	Cell Signaling Technology	Clone 20E3; Cat. 9718	Mouse and NMR
Ubiquitin	Cell Signaling Technology	Cat. 3933	Mouse and NMR

Supplementary Table S2. NMR CRISPR guide sequences

Guide ID	Sequence
sgM1	TGGGAGAGACCGGCGCACAG
sgM3	GTGCGTGTTTGTGCCTGTCC
sgM4	TGGGCTAGTGGGAACCTGCT
sgM5	CTAGTGGGAACCTGCTGGGG

Supplementary Table S3. Pharmacological agents used.

Compound	Company and Catalog Number	Treatment Concentration	Diluent
Bortezomib	Selleck Chemicals; S1013	Varied	DMSO
Cycloheximide	Sigma; 01810	100 μg/mL	Ethanol (200 proof)

Supplementary Fig. 1

A)

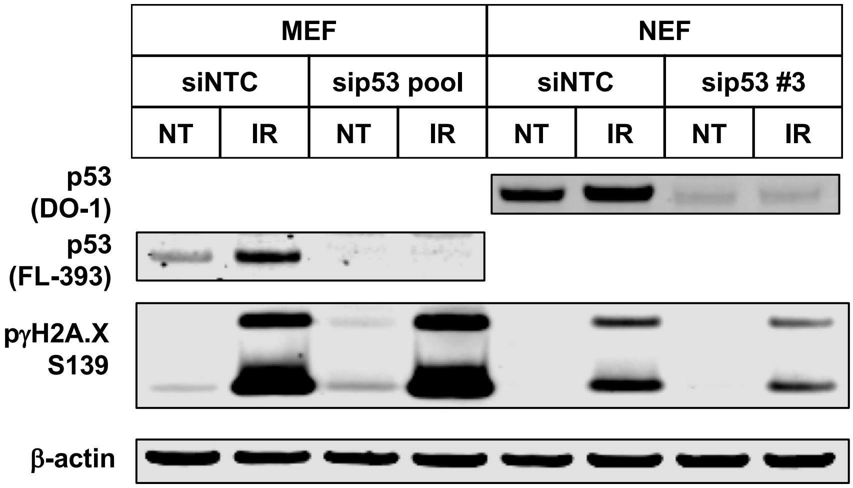

Supplementary Fig. 2

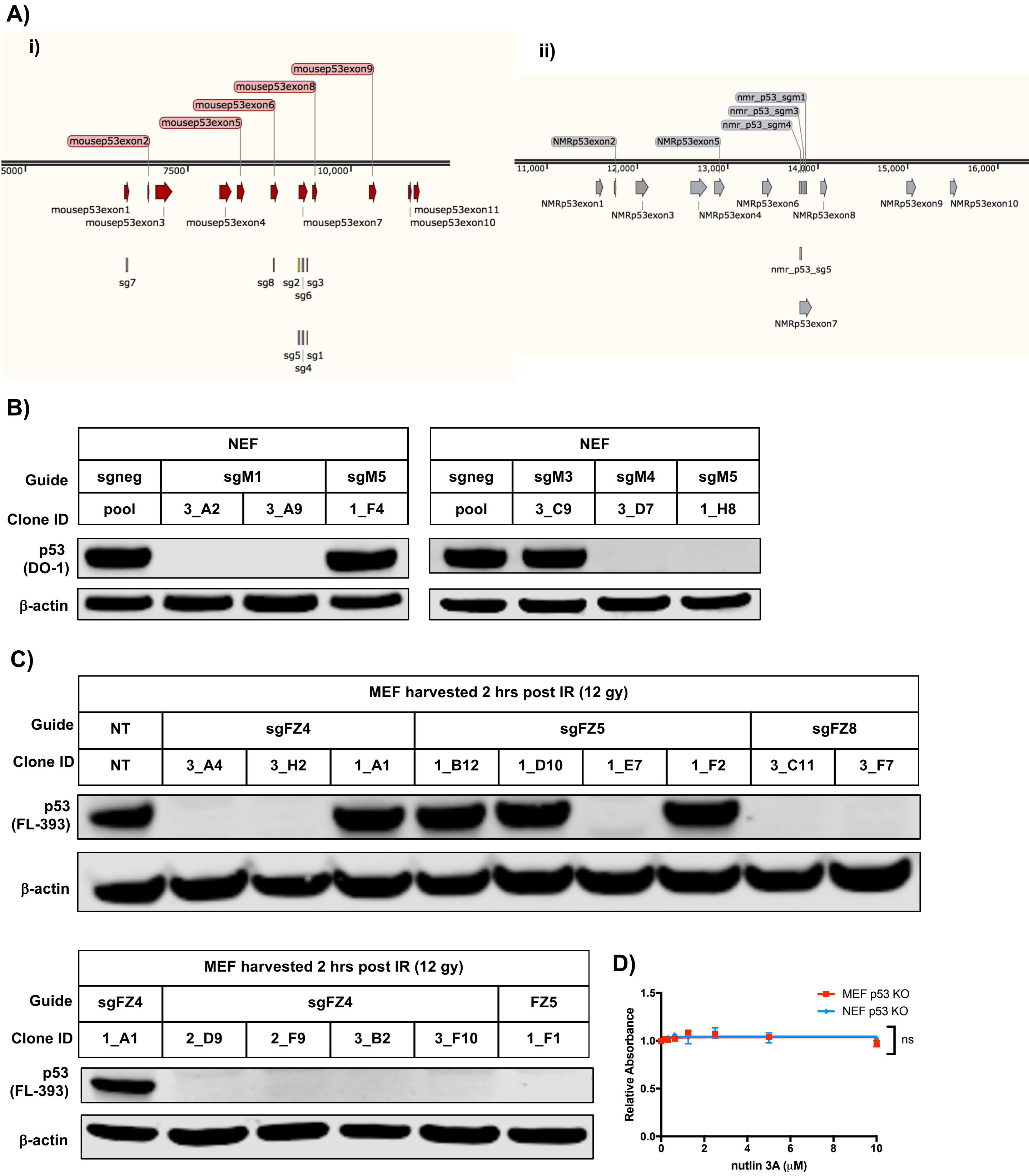

Supplementary Fig. 3

A)

Query: NMR p53

Subject: mouse p53 isoform a

cellular tumor antigen p53 isoform a [Mus musculus]

Sequence ID: NP_035770.2 **Length:** 390 **Number of Matches:** 1

See 9 more title(s)

Range 1: 4 to 390 GenPept Graphics Next Match Previous Match

Score	Expect	Method	Identities	Positives	Gaps
605 bits(1561)	0.0	Compositional matrix adjust.	305/391(78%)	327/391(83%)	4/391(1%)

```
Query  1    MEEPQSDLSIEPPLSQETFSDLWKLLPENNVLSSSLSSPMDDLLLSPEDVVNWLGGNPDE  60
            MEE QSD+S+E PLSQETFS LWKLLP  ++L S     MDDLLL P+DV  +  G P E
Sbjct  4    MEESQSDISLELPLSQETFSGLWKLLPPEDILPSPHC--MDDLLL-PQDVEEFFEG-PSE  59

Query  61   DVQVSAAPVPEPPTPVAPAPAAPAPATSWPLSSSVPSHKTYQGNYGFHLGFLQSGTAKSV  120
             ++VS AP  + P     P P APAPAT WPLSS VPS KTYQGNYGFHLGFLQSGTAKSV
Sbjct  60   ALRVSGAPAAQDPVTETPGPVAPAPATPWPLSSFVPSQKTYQGNYGFHLGFLQSGTAKSV  119

Query  121  TCTYSPVLNKLFCQLAKTCPVQVWVESPPPPGTRVRAMAIYKKSQHMTEVVRRCPHHERC  180
             CTYSP LNKLFCQLAKTCPVQ+WV + PP G+RVRAMAIYKKSQHMTEVVRRCPHHERC
Sbjct  120  MCTYSPPLNKLFCQLAKTCPVQLWVSATPPAGSRVRAMAIYKKSQHMTEVVRRCPHHERC  179

Query  181  SDSDGLAPPQHLIRVEGNLRAEYLDDRTTFRHSVVVPYDLPEVGSDCTTIHYNYMCNSSC  240
            SD DGLAPPQHLIRVEGNL  EYL+DR TFRHSVVVPY+ PE GS+ TTIHY YMCNSSC
Sbjct  180  SDGDGLAPPQHLIRVEGNLYPEYLEDRQTFRHSVVVPYEPPEAGSEYTTIHYKYMCNSSC  239

Query  241  MGGMNRRPILTIITLEDSSGNLLGRNSFEVRVCACPGRDRRTEEENFHKKGGSCPEPTPG  300
            MGGMNRRPILTIITLEDSSGNLLGR+SFEVRVCACPGRDRRTEEENF KK   CPE  PG
Sbjct  240  MGGMNRRPILTIITLEDSSGNLLGRDSFEVRVCACPGRDRRTEEENFRKKEVLCPELPPG  299

Query  301  SIKRALPTGTNSSPQPKKKPLDGEYFTLKIRGRERFEMFRELNEALELKDAQTEKEPGES  360
            S KRALPT T++SP  KKKPLDGEYFTLKIRGR+RFEMFRELNEALELKDA   +E G+S
Sbjct  300  SAKRALPTCTSASPPQKKKPLDGEYFTLKIRGRKRFEMFRELNEALELKDAHATEESGDS  359

Query  361  RPHSSYLKSKKGQSTSCHKKLMFKKEGPDSD  391
            R HSSYLK+KKGQSTS HKK M KK GPDSD
Sbjct  360  RAHSSYLKTKKGQSTSRHKKTMVKKVGPDSD  390
```

B)

Query: NMR p53

Subject: human p53

Tumor protein p53 [Homo sapiens]

Sequence ID: AAH03596.1 **Length:** 393 **Number of Matches:** 1

See 2 more title(s)

Range 1: 1 to 393 GenPept Graphics Next Match Previous Match

Score	Expect	Method	Identities	Positives	Gaps
620 bits(1600)	0.0	Compositional matrix adjust.	325/393(83%)	341/393(86%)	2/393(0%)

```
Query  1    MEEPQSDLSIEPPLSQETFSDLWKLLPENNVLSSSLSSPMDDLLLSPEDVVNWLGGNP--  58
            MEEPQSD S+EPPLSQETFSDLWKLLPENNVLS   S  MDDL+LSP+D+  W   +P
Sbjct  1    MEEPQSDPSVEPPLSQETFSDLWKLLPENNVLSPLPSQAMDDLMLSPDDIEQWFTEDPGP  60

Query  59   DEDVQVSAAPVPEPPTPVAPAPAAPAPATSWPLSSSVPSHKTYQGNYGFHLGFLQSGTAK  118
            DE  ++  A     P P AP PAAPAPA SWPLSSSVPS KTYQG+YGF LGFL SGTAK
Sbjct  61   DEAPRMPEAAPRVAPAPAAPTPAAPAPAPSWPLSSSVPSQKTYQGSYGFRLGFLHSGTAK  120

Query  119  SVTCTYSPVLNKLFCQLAKTCPVQVWVESPPPPGTRVRAMAIYKKSQHMTEVVRRCPHHE  178
            SVTCTYSP LNK+FCQLAKTCPVQ+WV+S PPPGTRVRAMAIYK+SQHMTEVVRRCPHHE
Sbjct  121  SVTCTYSPALNKMFCQLAKTCPVQLWVDSTPPPGTRVRAMAIYKQSQHMTEVVRRCPHHE  180

Query  179  RCSDSDGLAPPQHLIRVEGNLRAEYLDDRTTFRHSVVVPYDLPEVGSDCTTIHYNYMCNS  238
            RCSDSDGLAPPQHLIRVEGNLR EYLDDR TFRHSVVVPY+ PEVGSDCTTIHYNYMCNS
Sbjct  181  RCSDSDGLAPPQHLIRVEGNLRVEYLDDRNTFRHSVVVPYEPPEVGSDCTTIHYNYMCNS  240

Query  239  SCMGGMNRRPILTIITLEDSSGNLLGRNSFEVRVCACPGRDRRTEEENFHKKGGSCPEPT  298
            SCMGGMNRRPILTIITLEDSSGNLLGRNSFEVRVCAC GRDRRTEEEN  KKG    E
Sbjct  241  SCMGGMNRRPILTIITLEDSSGNLLGRNSFEVRVCACAGRDRRTEEENLRKKGEPHHELP  300

Query  299  PGSIKRALPTGTNSSPQPKKKPLDGEYFTLKIRGRERFEMFRELNEALELKDAQTEKEPG  358
            PGS KRALP  T+SSPQPKKKPLDGEYFTL+IRGRERFEMFRELNEALELKDAQ  KEPG
Sbjct  301  PGSTKRALPNNTSSSPQPKKKPLDGEYFTLQIRGRERFEMFRELNEALELKDAQAGKEPG  360

Query  359  ESRPHSSYLKSKKGQSTSCHKKLMFKKEGPDSD  391
             SR HSS+LKSKKGQSTS HKKLMFK EGPDSD
Sbjct  361  GSRAHSSHLKSKKGQSTSRHKKLMFKTEGPDSD  393
```

Supplementary Fig. S4A: Uncropped membranes from Fig. 1

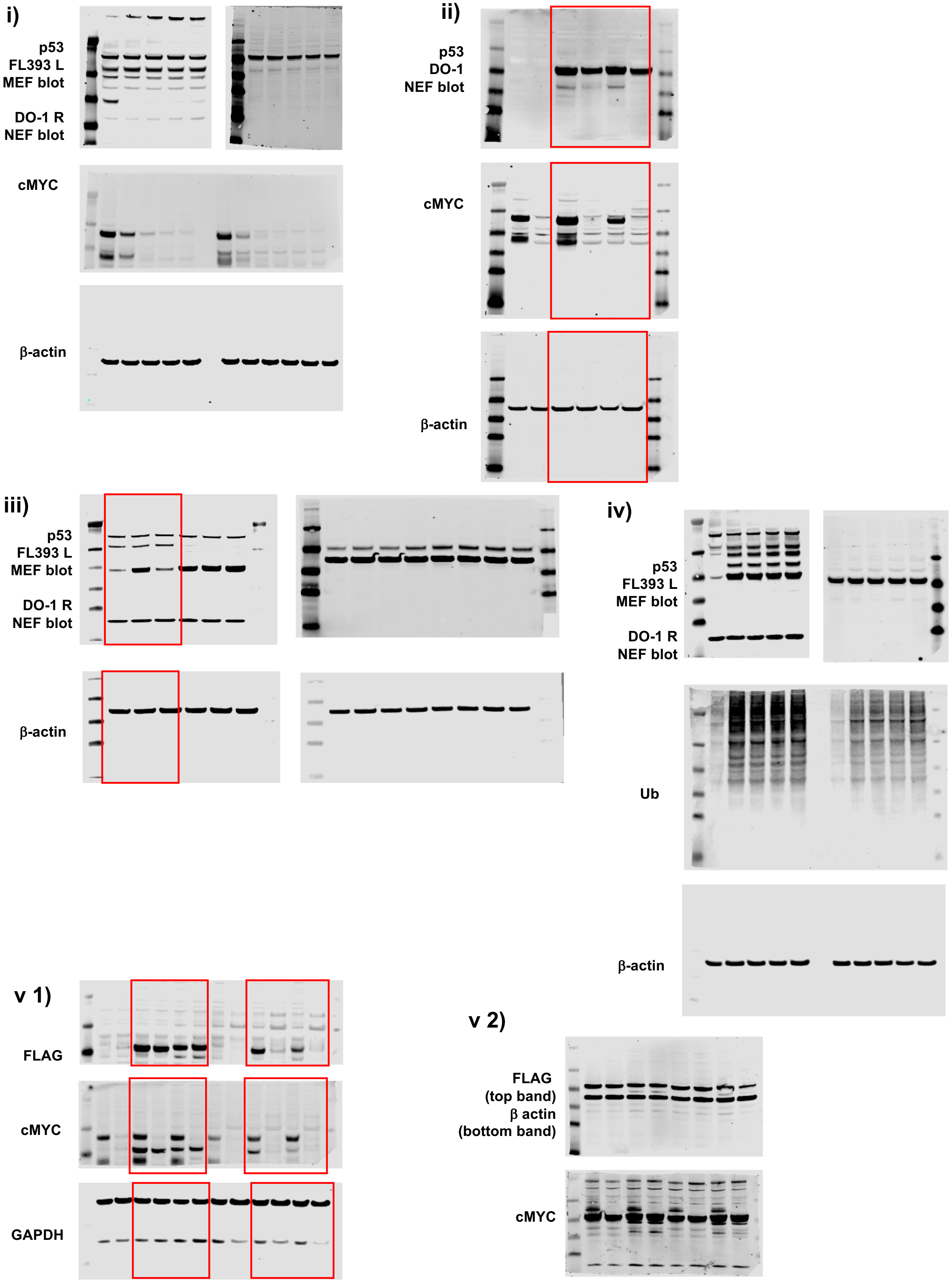

Supplementary Fig. S4B: Uncropped membranes from Fig. 2

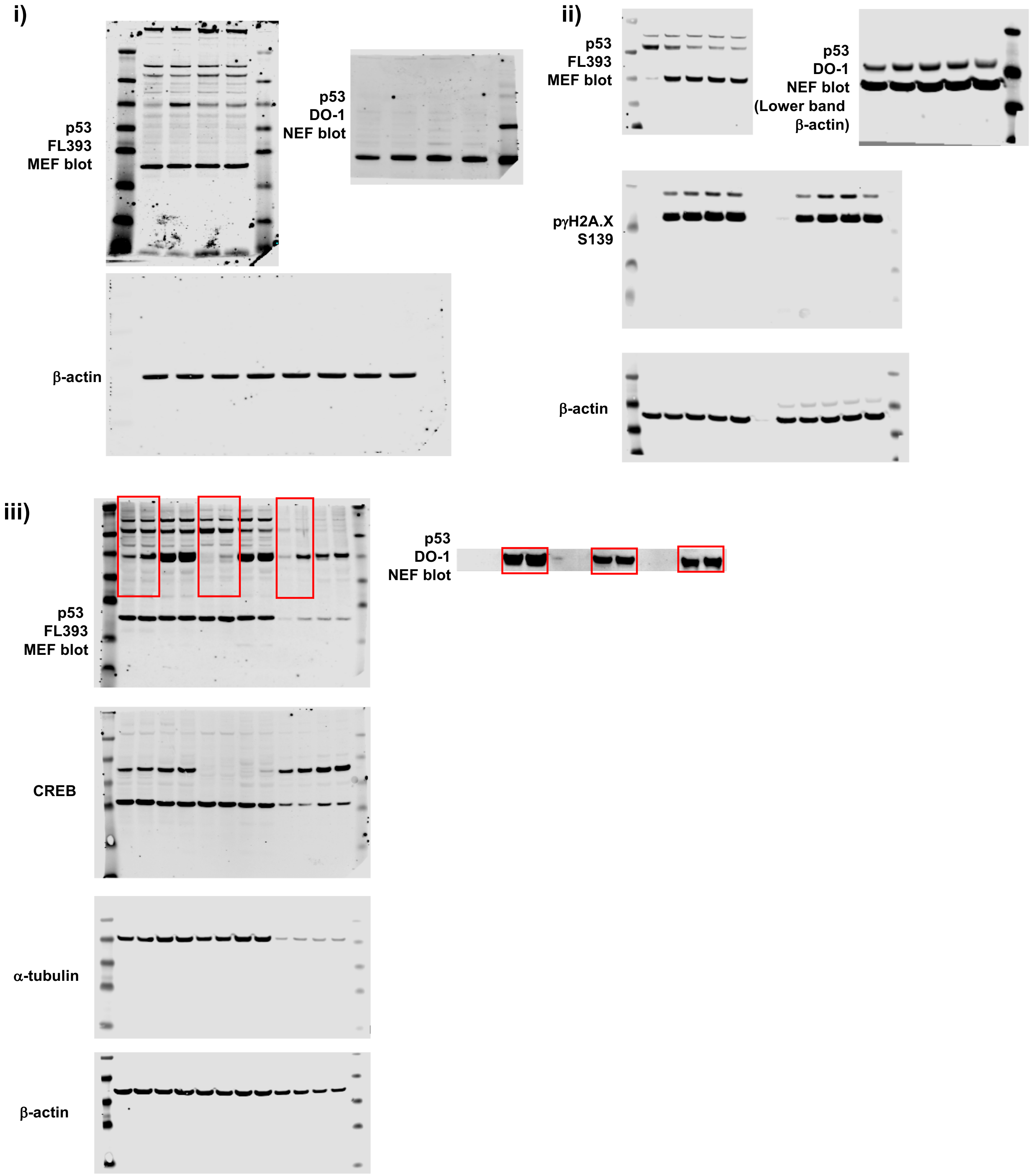

Supplementary Fig. S4C: Uncropped membranes from Fig. 3

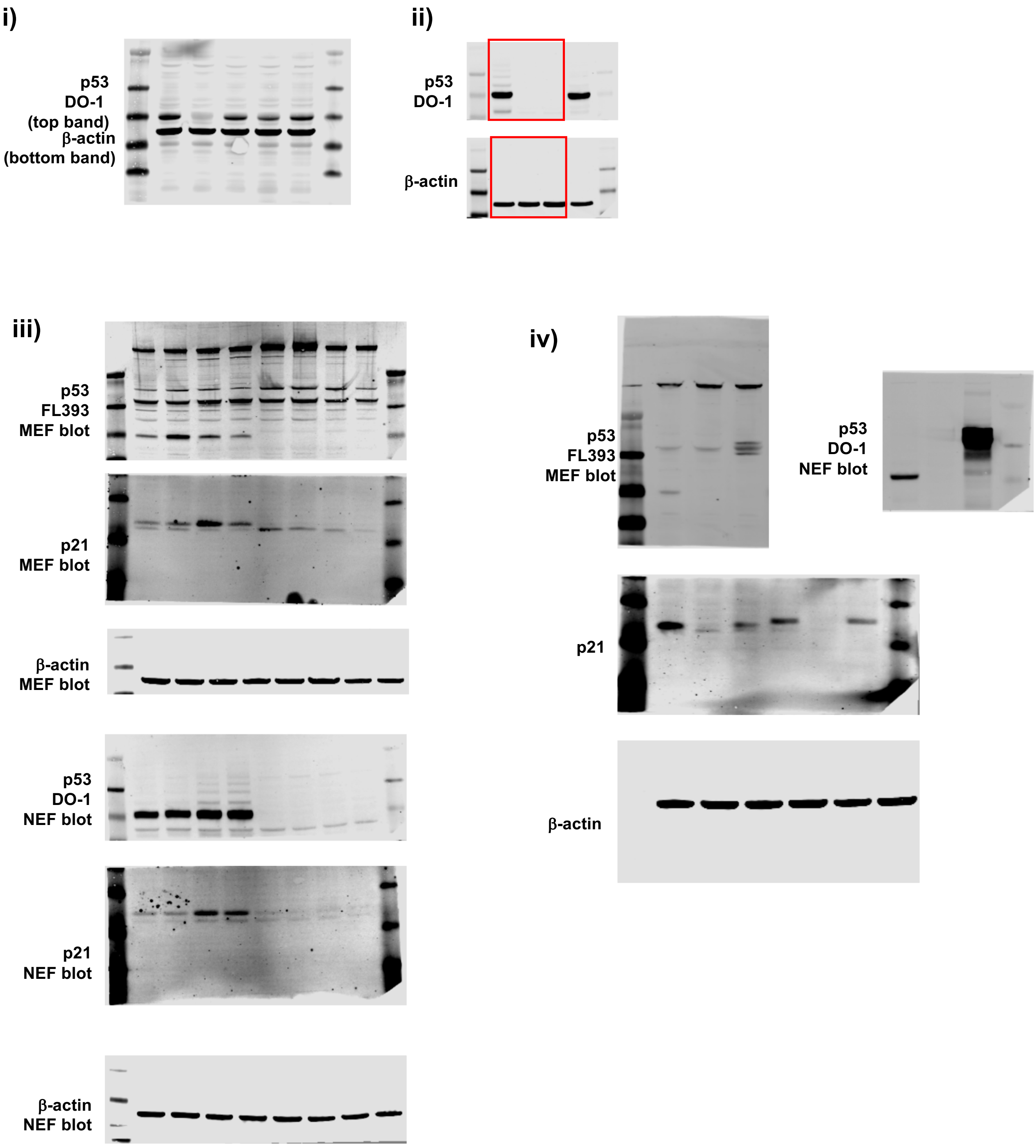

Supplementary Fig. S4D: Uncropped membranes from Supplementary Fig. S1

p53
FL-393
MEF blot

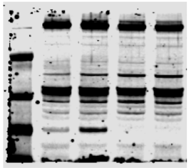

p53
DO-1
NEF blot

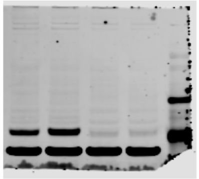

pγH2A.X
S139

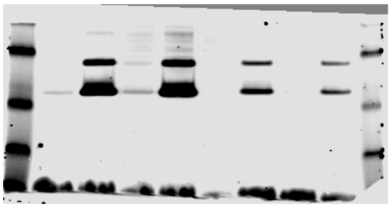

β-actin

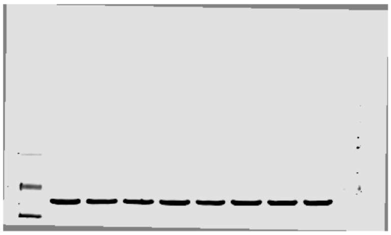

Supplementary Fig. S4E: Uncropped membranes from Supplementary Fig. S2

i)

p53
DO-1

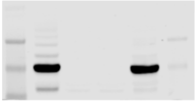

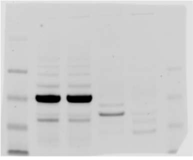

β-actin

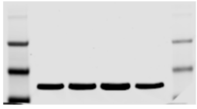

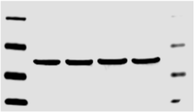

ii)

p53
FL-393

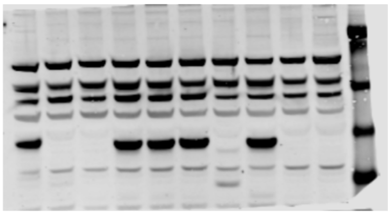

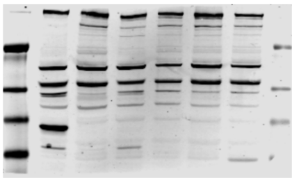

β-actin

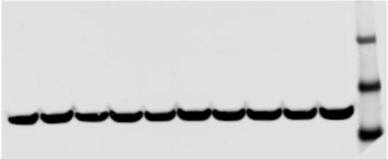

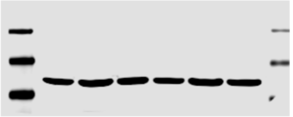

Supplement: Supplementary file 1 — Supplementary Information. [file 41598_2020_64009_MOESM1_ESM.pdf]
